# β-Neurexin Is a Ligand for the *Staphylococcus aureus* MSCRAMM SdrC

**DOI:** 10.1371/journal.ppat.1000726

**Published:** 2010-01-15

**Authors:** E. Magda Barbu, Vannakambadi K. Ganesh, Shivasankarappa Gurusiddappa, R. Chris Mackenzie, Timothy J. Foster, Thomas C. Sudhof, Magnus Höök

**Affiliations:** 1 Center for Infectious & Inflammatory Diseases, Institute of Biosciences and Technology, Texas A&M Health Science Center, Houston, Texas, United States of America; 2 Microbiology and Molecular Genetics Department, University of Texas Health Science Center at Houston, Houston, Texas, United States of America; 3 Moyne Institute of Preventive Medicine, Department of Microbiology, Trinity College, Dublin, Ireland; 4 Neuroscience Institute, Department of Molecular and Cellular Physiology, Stanford University, Palo Alto, California, United States of America; Dartmouth Medical School, United States of America

## Abstract

Gram-positive bacteria contain a family of surface proteins that are covalently anchored to the cell wall of the organism. These cell-wall anchored (CWA) proteins appear to play key roles in the interactions between pathogenic organisms and the host. A subfamily of the CWA has a common structural organization with multiple domains adopting characteristic IgG-like folds. The identified microbial surface components recognizing adhesive matrix molecules (MSCRAMMs) belong to this subfamily, as does SdrC from *S. aureus*. However, an interactive host ligand for the putative MSCRAMM SdrC was not previously identified. We have screened a phage display peptide library and identified a peptide sequence found in β-neurexin that binds SdrC. A synthetic peptide corresponding to the identified sequence as well as a recombinant form of the β-neurexin 1 exodomain binds SdrC with high affinity and specificity. Furthermore, expression of SdrC on bacteria greatly enhances microbial adherence to cultured mammalian cells expressing β-neurexin on their surface. Taken together, our experimental results demonstrate that β-neurexin is a ligand for SdrC. This interaction involves a specific sequence located in the N-terminal region of the mammalian protein and the N_2_N_3_ domain of the MSCRAMM. The fact that these two proteins interact when expressed on the appropriate cells demonstrates the functionality of the interaction. Possible implications of this interaction are discussed.

## Author Summary

*Staphylococcus aureus* is an opportunistic pathogen, distinguished by its potential to cause serious and life-threatening infections in animals and humans. The ability of this bacterium to adhere to host tissues is considered an early, essential event in the disease process and contributes to the success of the organism as a pathogen. Adherence to host tissues is mediated by a subfamily of cell-wall anchored proteins named MSCRAMMs (microbial surface component recognizing adhesive matrix molecules). Work in our laboratory suggested that many of these proteins share a common ligand binding mechanism targeting linear amino acid sequences. Based on these observations, we hypothesized that screening a phage display library of random peptides may identify receptors for MSCRAMMs. Using this method, we demonstrate that the putative MSCRAMM SdrC recognizes a sequence in the neuronal protein β-neurexin. Furthermore, we show that intact β-neurexin 1 is a functional ligand for the *S. aureus* MSCRAMM SdrC. Successful implementation of this approach may open avenues for the identification of additional host ligands and the design of anti-staphylococcal therapeutics able to inhibit these interactions.

## Introduction

The Gram-positive opportunistic pathogen *Staphylococcus aureus* can cause a spectrum of infections in humans and animals that differ in severity. Some relatively minor skin infections, such as folliculitis, impetigo and cellulitis, can progress to life threatening diseases like sepsis, endocardatis, osteomylitis and pneumonia [1].

The molecular pathogenic mechanisms of different staphylococcal infections are incompletely understood but studies suggested that a critical factor for the pathogenic success of this organism depends on its ability to adhere effectively to multiple host tissues [2],[3],[4]. The adhesins mediating staphylococcal adherence and colonization often target the extracellular matrix of the host and hence belong to the MSCRAMM family [4]. In Gram-positive bacteria, many MSCRAMMs are cell-wall-anchored (CWA) proteins [5],[6] with a similar structural organization [7]. These proteins contain an amino terminal signal sequence followed by an A-region that often harbors the ligand-binding sites. The A-region is comprised of sub-domains (called N-domains) adopting an immunoglobulin G-like (IgG-like) fold [8]. Sometimes the A-region is followed by a B-region containing repeated β-sandwich modules of unknown function. In the case of the Sdr-subfamily of staphylococcal MSCRAMMs, the B-region is accompanied by a repeat (R) domain composed of multiple Ser-Asp dipeptide repeats (SD-repeat or Sdr) (reviewed in [9]).

The ligand-binding activity of SdrG, a fibrinogen (Fg)-binding MSCRAMM of *Staphylococcus epidermidis* [10], was shown to proceed via a “dock, lock and latch” mechanism [7],[11]. A crystal structure of a Fg-based peptide in complex with the SdrG_N2N3_ domain suggested that the peptide “docks” into the groove formed between the N2 and N3 domain. Upon binding the C-terminal extension of the N3 domain is redirected to cover and “lock” the ligand peptide in place and further stabilizes the complex by complementing a β-sheet in the N2 domain, thus functionally serving as a “latch” [11]. The back of the latching trench contains a motif, TYTFTDYVD, conserved in several other staphylococcal MSCRAMMs. Genome-based bioinformatics of five Gram-positive bacterial strains revealed that all organisms contain proteins with predicted IgG-like folded domains, an LPXTG-motif and a TYTFTDYVD-motif. Therefore, the “dock, lock and latch” mechanism was proposed as a common ligand binding strategy among these proteins.

In the current study, we used SdrC as bait for screening a phage library displaying 12-mer linear peptides (New England Biolabs Ph.D.™ -12 Phage Display Peptide Library). The *sdrC* gene is the first gene in a bi- or tri-partite gene cluster that also contains either *sdrD* or *sdrD* and *sdrE*. The predicted domain organization of SdrC is similar to that of SdrG with the N2 and N3 sub-domains of the A-region adopting an IgG-like fold [12],[13].

The phage display strategy, used in this study, identified beta (β) neurexin as a ligand for the orphan MSCRAMM SdrC. Subsequent biochemical and cell biology experiments confirmed that β-neurexin is a ligand for SdrC.

Neurexins are neuronal cell adhesion molecules that interact with neuroligins and appear to play an important role in synapse function (reviewed in [14]). Previously, the neurexins were discovered as the receptor for the neurotoxin α-latrotoxin of the black-widow spider [15].

## Results

### Validation of the phage display method to identify MSCRAMM ligands

Analysis of peptides or protein segments displaced on the surface of phage is a useful method to identify partners in protein-protein interaction systems [16],[17],[18],[19]. To determine if this method can be used to identify ligands for MSCRAMMs from Gram-positive bacteria, we performed a validation experiment using SdrG_N2N3_ as bait in a screen of a commercially available 12-mer linear library (New England Biolabs Ph.D.-12 Phage Display Peptide Library). We carried out three successive rounds of panning against immobilized SdrG_N2N3._ To confirm that the enrichment of the phage clones between panning rounds was the result of specific interactions, the phage pool before and after each step of amplification was also tested for binding to BSA. We found that the number of phage binding to SdrG increased relative to the number of phage binding to BSA (Table 1) suggesting a specific enrichment for SdrG. We then sequenced the DNA purified from ten phage plaques obtained after the third round of panning. Alignment of the inserted amino acid sequences revealed FSARG as a consensus in the majority of the clones (Table 2). This result was consistent with previous results from our laboratory identifying FFSARG as the binding motif in Fg for SdrG [7]. The successful identification of the SdrG binding sequence suggested that phage display of peptide libraries could be used to find binding partners for orphan MSCRAMMs.

**Table 1:** Number of transducing units (TUs) obtained after each panning against BSA and SdrG_N2N3_.

Panning*	Ligand	Total bound TUs
1	BSA	1×10^4^
	SdrG	4×10^5^
2	BSA	1×10^2^
	SdrG	3×10^7^
3	BSA	10
	SdrG	2×10^8^

*Number of input phage was 1.5×10^11^ for each panning.

**Table 2:** Peptide motifs binding to SdrG_N2N3_.

Peptide sequence
VYPTLHFSARGSG
QSQWPVLFFSARG
SNIPFQFSARGPG
SENLQFSARGPGG
HMEGSELSFSARG
AHQDETLAFSARG
NHPFNALSFSSRG
YADLLAQFSSKSG
YLPSQISAIGRAG
NPVIRYASRSHSG

*consensus sequence shown in bold.

### Identification of the putative ligand-binding domain of SdrC

Sequence alignments between the A-region of SdrC (SdrC_A_ or SdrC_52-496_) and previously identified staphylococcal MSCRAMMs revealed a modest identity (less than 20%). In contrast, a comparison of the predicted structure of SdrC_A_ with the determined structures of the crystallized staphylococcal MSCRAMMs ClfA and SdrG pointed to close structural similarities [7],[20],[21]. A sub-segment of the SdrC A-region corresponding to residues 178–496 was predicted by PHYRE (http://www.sbg.bio.ic.ac.uk/phyre/) to adopt a structure similar to those determined for the N2N3 sub-domain of the Fg-binding MSCRAMMs ClfA (PHYRE e = 3.1×10^−32^) and SdrG (PHYRE e = 9.6 10^−32^). Similar to the N2N3 domains of ClfA and SdrG, SdrC_178–496_ likely contains two IgG-like folded domains and has a TYTFTDYVD-like “latching cleft” motif in the predicted N2 sub-domain. Therefore, we designated SdrC_178–496_ as the N2N3 domain and SdrC_52–177_ as the N1 domain.

**Figure 1:**
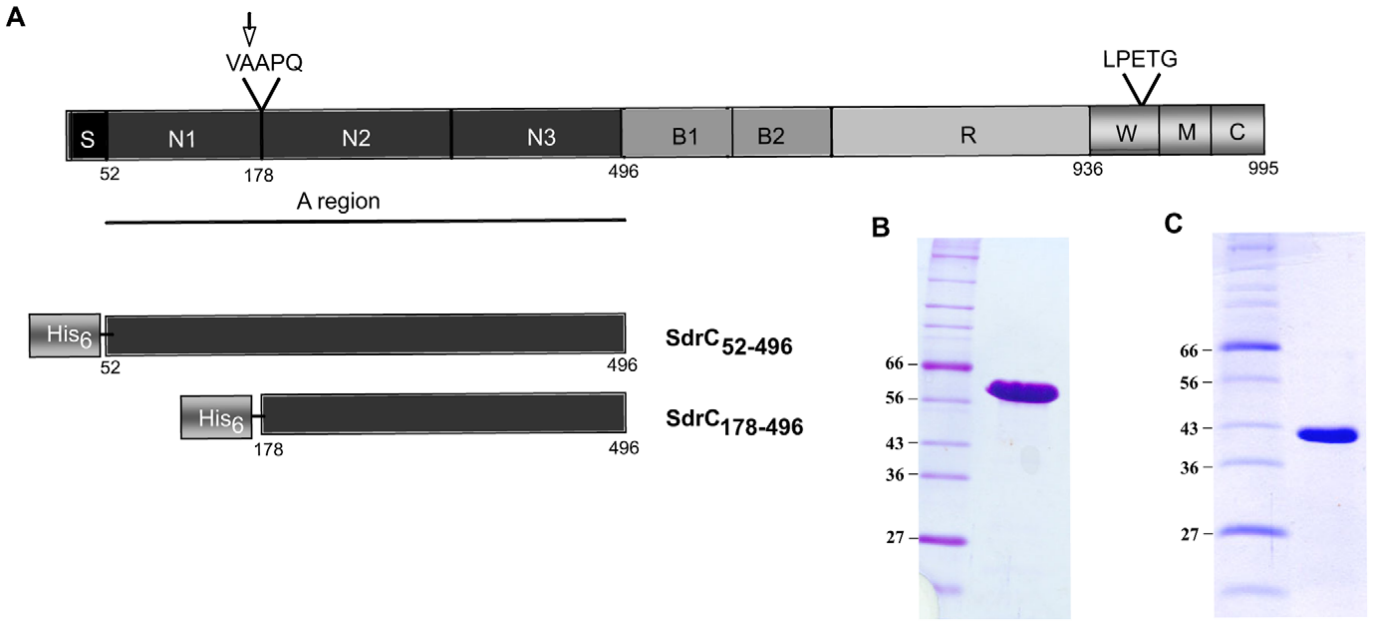
Structural organization of SdrC and recombinant proteins. (**A**) Schematic representation of SdrC domain structure. S, signal sequence; A region underlined composed of N1, N2 and N3, B1 and B2, B repeats, R, serine-aspartic acid repeat region; W, wall-spanning fragment; M, transmembrane domain; C, cytoplasmic tail; LPETG, cell wall anchoring motif; VAAPQ, cleavage site; the arrow indicated the cleavage position. Also shown, are the His-tag recombinant SdrC proteins used in this study. (**B**), Coomassie-stained SDS-PAGE gel of pure recombinant SdrC_A_. (**C**) Coomassie-stained SDS-PAGE gel of the recombinant SdrC_A_ after 2 weeks storage at 4°C.

Both SdrC_52–496_ (SdrC_A_) and SdrC_178–496_ (SdrC_N2N3_) were expressed with an N-terminal His tag in *E. coli* (Fig. 1A). SDS-PAGE analysis of purified recombinant proteins indicated apparent molecular weights of 60 KDa (Fig. 1B) and 45 KDa, respectively (Fig. 1C). Matrix-assisted laser desorption ionization mass spectrometry suggested that the masses of the purified recombinant proteins are close to the theoretical molecular masses calculated from the primary amino acid sequences (51,194 KDa compared to 50,924 KDa for SdrC_A_ and 38,358 KDa compared to 38,340 KDa for SdrC_N2N3_). Thus, the recombinant SdrC proteins migrate aberrantly on SDS-PAGE. Aberrant migration on SDS-PAGE is common for the recombinant A-region of MSCRAMMs and thought to be due to their hydrophilic index [10],[12],[22]. The recombinant A-region was quickly degraded to a single proteolytically stable segment with an apparent molecular mass of 45 KDa. The N-terminal fragment released was apparently further degraded and was rarely detected on SDS-PAGE. N-terminal sequence analysis of the truncated, resistant segment revealed that cleavage had occurred between Ala^177^ and Ala^178^. Mass spectrometry indicated a molecular weight of 38,358 KDa compared with 38,340 KDa calculated from the predicted amino acid sequence of the stable fragment suggesting that the cleavage event occurred only at the N-terminus of the A-region. Cleavage of the N-terminal sub-segment (N1) of the A-region has also been observed with other recombinant MSCRAMMs (ClfA, ClfB, SdrG) and may be explained by disordered segments in the N1 sub-domains as well as the presence of minute amounts of contaminating proteases in the MSCRAMM preparations [7],[23],[24]. The proteolysis resistant fragment of SdrC_A_ corresponds to the tandem IgG-like domains (N2N3) as predicted by PHYRE (http://www.sbg.bio.ic.ac.uk/phyre/).

### Identification of β-neurexin as a potential binding partner for SdrC_N2N3_ using phage display

**Figure 2:**
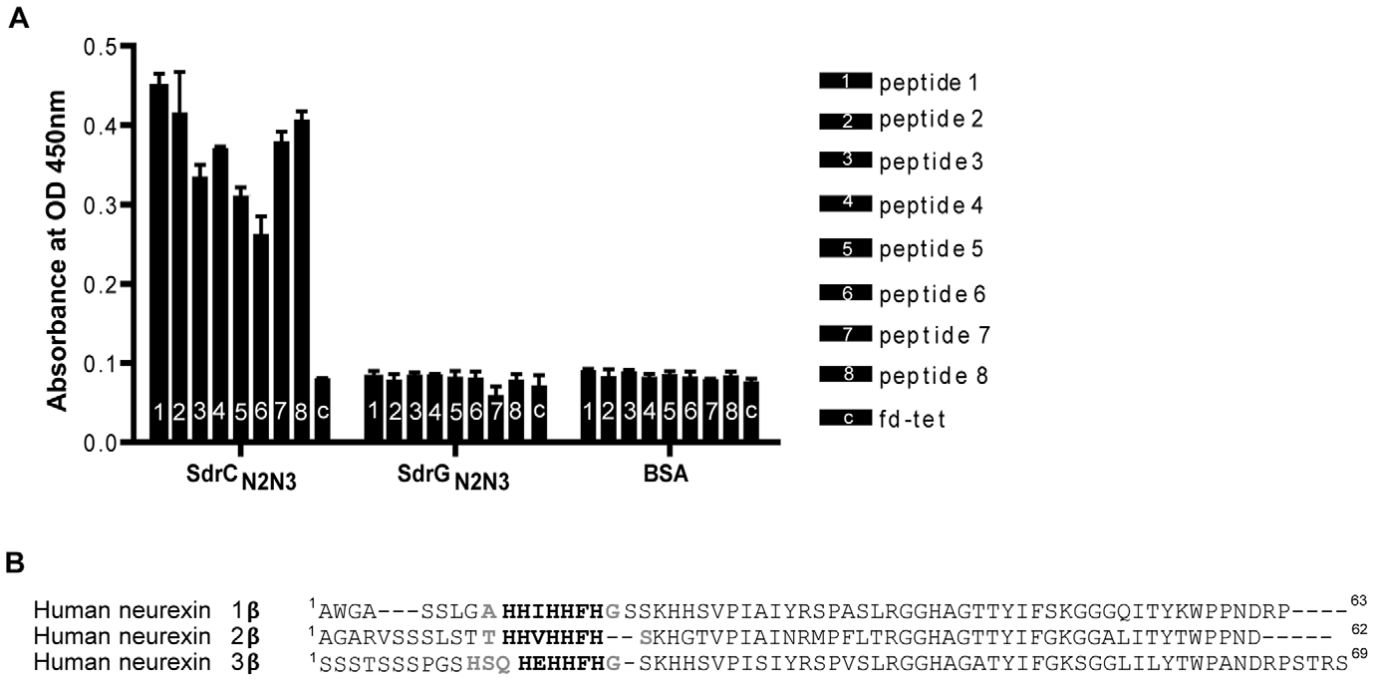
Identification of β-neurexins as the potential SdrC-binding partner. (**A**), Phage obtained after the third panning round bound specifically to SdrC_N2N3_. Fifty random phage clones obtained after the 3^rd^ round of panning were incubated with immobilized SdrC_N2N3_, SdrG_N2N3_ and BSA. Shown are the eight phage clones with the highest binding affinity (*p<0.001) for SdrC_N2N3_ in comparison with SdrG_N2N3_, BSA or fd-tet (insertless phage). (**B**), Consensus sequence search. Pattern searches with the degenerated consensus (P,T,A)HH(I,M)HHFH(G,R,S,Q,T,A) against a human protein database returned β-neurexin isoforms as SdrC-ligands. Consistent residues of the consensus are highlighted in bold, variable residues are highlighted in gray.

We screened the New England Biolabs Ph.D.-12 Phage Display Peptide Library using immobilized recombinant SdrC_N2N3_ as bait. After the 3^rd^ round of panning, fifty randomly picked plaques were selected and the corresponding phages were screened again for binding to SdrC_N2N3_, to SdrG_N2N3_ and to BSA as controls. All clones bound to SdrC, however, phage from eight clones displayed significantly higher binding to SdrC_N2N3_ compared to SdrG_N2N3_ and BSA (Fig. 2A). The clone inserts were sequenced and an alignment of the deduced amino acid sequences is shown in Table 3. From these sequences we could identify a degenerate consensus sequence (P,T,A)HH(I,M)HHFH(G,R,S,Q,T,A) which was then used for a pattern search of the human protein database employing an algorithm allowing for zero, one or two residue mismatches. Zero mismatches returned only neurexin 1β, one mismatch returned neurexin 1β and neurexin 2β and two mismatches returned neurexin 1β, neurexin 2β, neurexin 3β and a T-type voltage-dependent calcium channel (Table S1). All proteins returned by the search were screened for the presence of the consensus sequence within the extracellular domain of the protein. As shown in Fig. 2B, an alignment of the N-terminal extracellular segments of the β-neurexin isoforms revealed variations of the consensus sequence identified by phage display. In addition, the peptide sequence displayed by phage number one confirms a sequence found in neurexin 1β and the sequenced displayed by phage eight is very similar to a sequence found in neurexin 3β [25].

**Table 3:** Peptide motifs binding to SdrC_N2N3_.

	Peptide sequence*
**Peptide 1**	HWRTHHIHHFHQG
**Peptide 2**	MSPHHMHHSHGHG
**Peptide 3**	AKLAHHIHHFHGG
**Peptide 4**	WVPHHIHHFHRAG
**Peptide 5**	YTHHHHSWRLHTG
**Peptide 6**	AHHPHAWRHSHKG
**Peptide 7**	STFHFHTHKARHG
**Peptide 8**	HSQHHRFHHTYPG

*consensus sequence shown in bold.

The amino-terminal segments of the β-neurexins contain an unusually long signal sequence (∼50 aa), a neuroligin binding site and a glycosylation site in close proximity to the transmembrane domain. The C-terminal cytoplasmic segment contains a PDZ domain presumably involved in intracellular signaling [25]. The putative SdrC binding site identified by phage display corresponds to amino acid residues 10 to 16 and maps in the neuroligin-binding domain exposed on the cell surface.

### Recombinant SdrC_N2N3_ binds recombinant neurexin 1β (Nrx1β)

**Figure 3:**
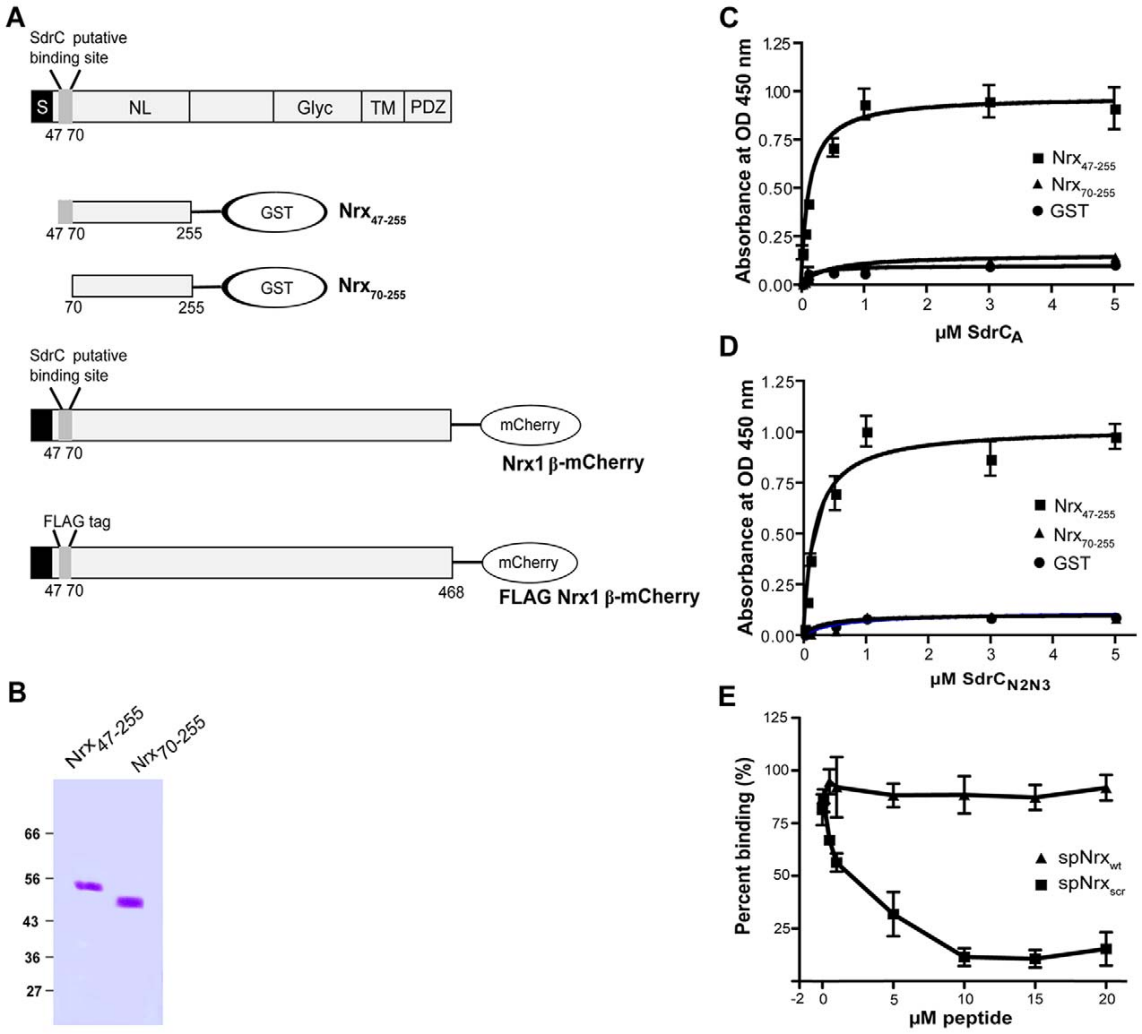
Structural organization of β-neurexins. (**A**) A cartoon representation of β-neurexin domain structure. S, signal sequence; NL, neuroligin-binding domain; Glyc, glycosylation domain; TM, transmembrane domain; PDZ, intracellular signaling domain. Also shown, are the recombinant proteins used in this study. (**B**) Coomassie-stained SDS-PAGE gel of GST-tagged neurexin 1β domains recombinant proteins. Increasing concentrations of SdrC_A_ (**C**) and SdrC_N2N3_ (**D**) were incubated with immobilized Nrx_47-255_ (black square), Nrx_70–255_ (black triangle) or GST (black circle) for 1 hour at room temperature. The apparent K_D_ (concentrations required for half maximum binding) values were 1.85±0.94×10^−7^ M for SdrC_A_ and 1.26±0.28×10^−7^ M for SdrC_N2N3_. (**E**) SdrC_178–496_ (0.5 µM) was incubated with increasing concentrations of spNrx_wt_ peptide (black square) or spNrx_scr_ peptide (black triangle) at room temperature. After 2 hours, the mixture was incubated with immobilized Nrx_47–255_. The values represented here are the mean±SD of triplicates from three experiments.

The phage display library screening identified beta neurexins as potential binding partners for SdrC. To explore a possible interaction between the recombinant forms of SdrC_N2N3_ and the exodomain of Nrx1β we expressed in *E. coli* two recombinant Nrx1β fragments with a C-terminal GST tag (Fig. 3A). The fragment designated Nrx_47-255_ corresponds to the first 208 amino acids residues of Nrx1β protein which contains the putative SdrC-binding site identified by phage display (amino acid residues 57 through 63). Nrx_70-255_ is a truncated version of the segment described above in which the putative SdrC-binding site was deleted (Fig. 3B). Both recombinant Nrx1β-GST proteins were immobilized on 96-multiwell plates and used in a solid phase binding assay. SdrC_A_ bound the immobilized Nrx_47-255_ in a concentration dependent manner but did not bind to either Nrx_70-255_ or the purified fusion partner GST (Fig. 3C). Similar results were obtained when the intact SdrC_N2N3_ region (Fig. 3D) or the proteolytically resistant sub-segment released from the recombinant SdrC_A_ (data not shown) were used as probes.

To further demonstrate that HHIHHFH is the specific binding site in Nrx1β for SdrC, we used a synthetic peptide (sp) with a sequence corresponding to the N-terminus of Nrx1β, SLGAHHIHHFHGSSKHHS (spNrx_wt_), in attempts to inhibit the binding between SdrC_N2N3_ and Nrx_47–255._ As a control we used a peptide with the same residues but where the sequence was scrambled to HSHIKLHSHGHSFGHA (spNrx_scr_). Recombinant SdrC_N2N3_ (0.5 µM) was incubated with increasing concentration of either peptide prior to being added to the Nrx coated plate. spNrx_wt_ inhibited the interaction between SdrC_N2N3_ and Nrx_47–255_ in a concentration dependent manner. We did not observe an inhibition of the MSCRAMM-β-neurexin interaction when spNrx_scr_ was used in the assay (Fig. 3E). The observed biding of recombinant SdrC to Nrx1β and the subsequent synthetic peptide inhibition assay suggests that the interaction between these proteins is specific and that the sequence motif identified in the phage display experiment represents the ligand-binding site in Nrx1β for SdrC.

### SdrC binds the Nrx_wt_ peptide with high affinity

**Figure 4:**
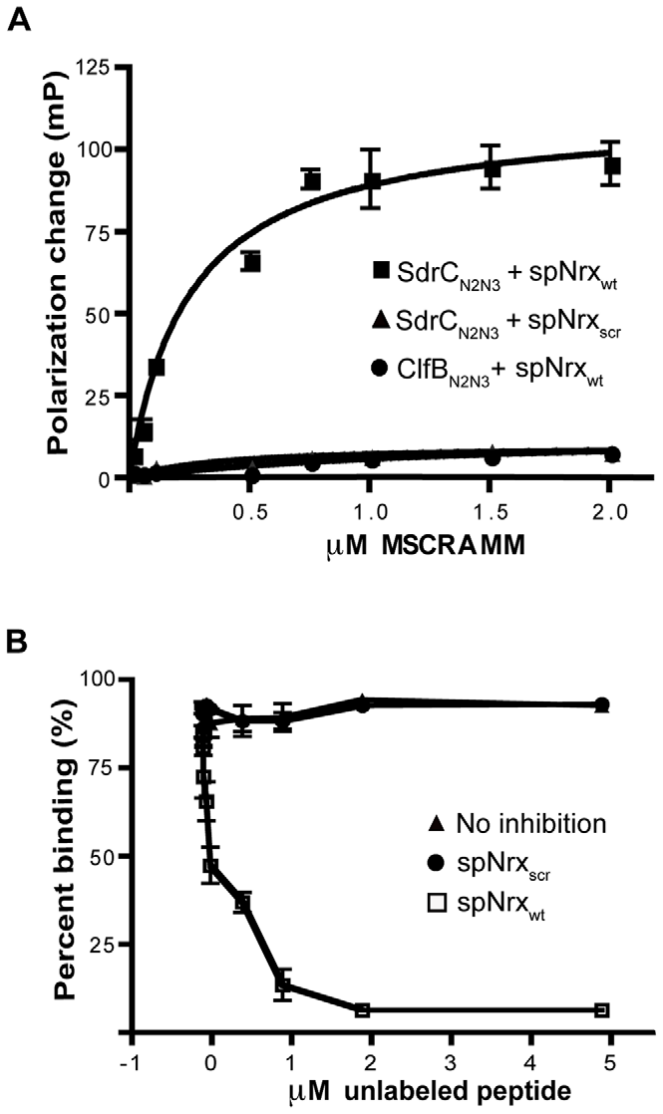
Determination of equilibrium constant of binding. (**A**) SdrC_N2N3_ binds specifically to β-neurexin 1. Increasing concentrations of SdrC_N2N3_ or ClfB_N2N3_ (black circle) were incubated with 10 nM fluorescein-labelled spNrx_wt_ peptide (black square) or spNrx_scr_ peptide (black triangle) for 3 hours at room temperature in the dark. Equation ΔP = ΔP_max_ [protein]/(K_D_+[protein]) was used to calculate the equilibrium constant. The values from three experiments returned a K_D_ = 2.50±0.49×10^−7^ M. (**B**) SdrC_N2N3_ (0.5 µM) (black triangle) was incubated with increasing concentration of unlabelled spNrx_wt_ peptide (white circle) or spNrx_scr_ peptide (black circle) at room temperature. After 3 hours, the mixture was incubated for another 3 hours at room temperature in the dark with 10 nM fluorescein labeled spNrx_wt_ peptide. Fluorescence polarization was determined as described above. The values represented here are the mean±SD of triplicates from three experiments.

We used fluorescence polarization to determine the dissociation constant for the SdrC_N2N3_-Nrx1β peptide interaction. spNrx_wt_ and spNrx_scr_ were labeled with fluorescein and incubated with increasing concentrations of recombinant MSCRAMM. SdrC_N2N3_ bound spNrx_wt_ in a concentration dependent, saturable manner with a K_D_ of 2.5±0.5×10^-7^ M. We did not detect a significant binding of SdrC_N2N3_ to the fluorescein-labeled spNrx_scr_. A control MSCRAMM, ClfB_N2N3_, did not bind to the fluorescein-labeled spNrx_wt_ (Fig. 4A).

To eliminate the possibility that the binding was dependent of the fluorescein label introduced in the wild-type peptide, we tested the ability of unlabeled spNrx_wt_ and spNrx_scr_ to inhibit the binding of SdrC_N2N3_ to fluorescein-labeled spNrx_wt_. Increasing concentrations of unlabeled peptides were incubated with SdrC_N2N3_ for three hours at room temperature before the fluorescent polarization experiment was performed. Fig. 4B shows that the unlabeled spNrx_wt_ but not the spNrx_scr_ inhibited the binding between SdrC_N2N3_ and the fluorescein-labeled spNrx_wt_. These results demonstrated that SdrC binds with relatively high affinity to the identified amino acid sequence motif in β-neurexin.

### SdrC mediates bacterial attachment to cells expressing Nrx1β

**Figure 5:**
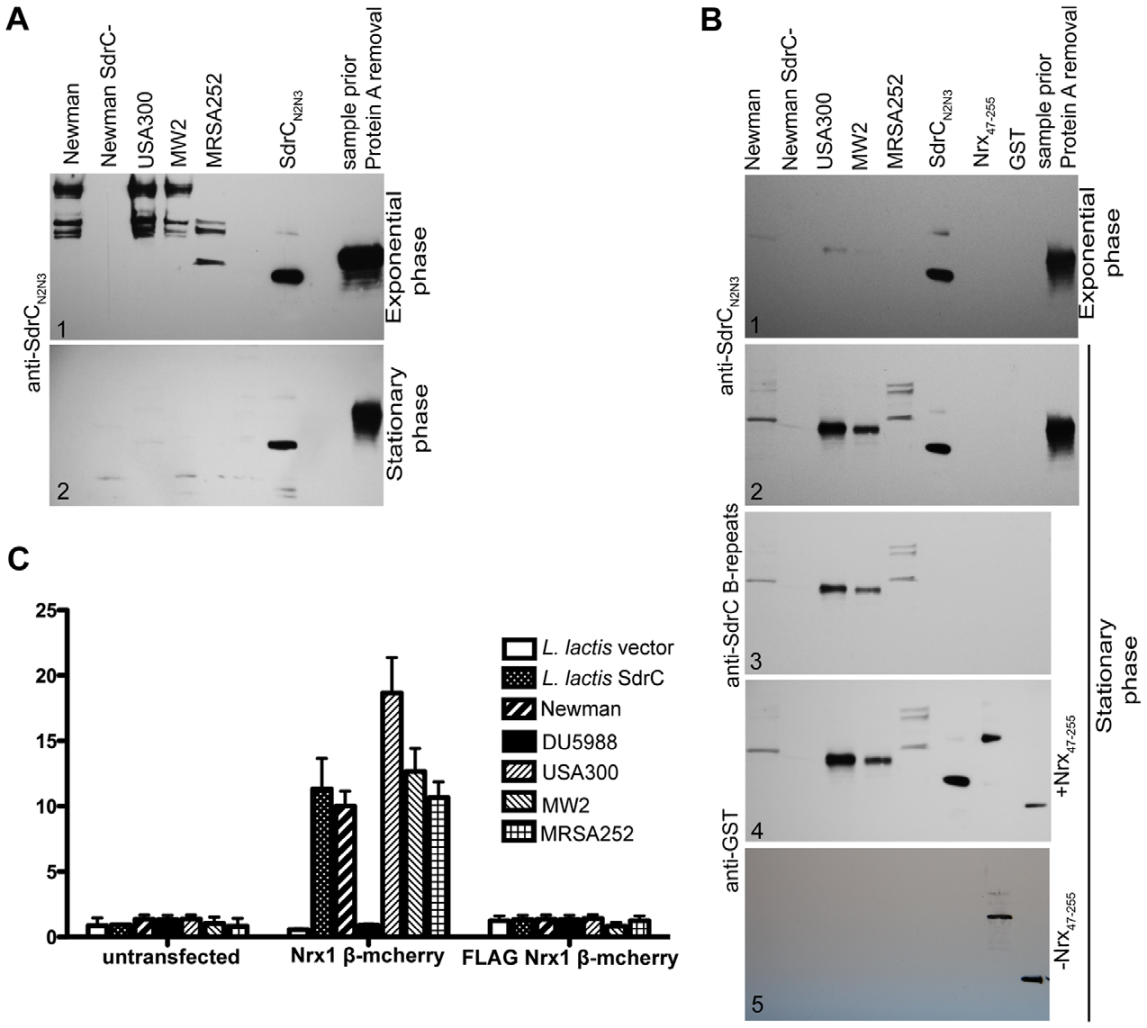
SdrC mediates *S. aureus* clinical isolates adherence to Nrxβ expressing cells. (**A**) Western immunobloting to detect SdrC expression on the surface of different bacterial strains. *S. aureus* Newman, DU5988 and clinical strains USA300, MW2, MRSA252 were grown to exponential phase (**A1**) or stationary phase (**A2**) in BHI. CWA proteins extracted as described in material and methods were separated on 4–15% gradient SDS-PAGE gradient gels, transferred to nitrocellulose and probed with anti-SdrC serum. (**B**) Culture supernatants from the above mentioned strains grown to exponential (**B1**) or stationary phase (**B2**) were concentrated and separated on 4–15% gradient SDS-PAGE gradient gels after protein A removal. After gel separation the proteins were transferred to nitrocellulose and probed with anti-SdrC serum (**B1** and **B2**). The nitrocellulose blot corresponding to the stationary phase supernatants was stripped and re-probed with anti-SdrC B-repeats antibody (**B3**). The same blot was stripped again and probed with Nrx_47-255_. The binding of Nrx1β was detected with anti-GST serum (**B4**). A second membrane (**B5**) was not incubated with Nrx_47-255_ and therefore served as control for the primary and secondary sera. (**C**) *L. lactis* empty vector, *L. lactis* SdrC, *S. aureus* Newman, DU5988 (*S. aureus* Newman *sdrC*::Em^r^), USA300, MW2 or MRSA252 were incubated with CHOK1 transiently transfected with Nrx1β-mCherry or FLAG Nrx1β-mCherry grown in 24 well plates. Attachment of bacteria was reported as a percentage of total bacteria at the end of incubation. Values shown represent mean±SD of three experiments (*p≤0.001).

Next we sought to determine if common clinical strains express SdrC and if the SdrC displayed by these isolates are capable of binding Nrx1β. Western blotting analysis of cell wall extractions from USA300, MW2 and MRSA252 display SdrC on their surface during exponential phase of growth in BHI (Fig. 5A1) and RPMI 10% FBS media, and when grown on TSB sheep blood agar (data not shown). SdrC was not found in cell wall extractions from cells in stationary phase of growth (Fig. 5A2). We reasoned that SdrC absence from the cell wall extractions in the late stages of growth might be due to transcription cessation and proteolytical release from the cell wall. Western blotting analysis of concentrated culture supernatants revealed that SdrC was not detected in the supernatant during exponential growth but that all strains tested released SdrC fragments during late stationary growth phase (Fig. 5B1 and Fig. 5B2). SdrC fragments released in the supernatant migrated slower than recombinant SdrC_N2N3_ on the SDS-PAGE gels suggesting that a larger fragment of the protein is released from the cell wall. Immunoblotting analysis demonstrated that these fragments were recognized by antibodies raised against recombinant N2N3 domains and B-repeats (Fig. 5B3). USA300, MW2 and Newman display and release similar SdrC segments whereas MRSA252 expresses different SdrC species. One possible explanation for this difference may lie in the differences between their amino acid sequences (Fig. S5). These fragments were probed in ligand affinity western blots with recombinant Nrx_47–255_ protein. Our data indicated that all strains tested release an active form of the SdrC protein capable to interact with Nrx1β (Fig. 5B4). A control membrane not probed with Nrx_47–255,_ did not indicate cross reactivity of anti-GST serum with SdrC fragments released into the supernant (Fig. 5B5).

We next tested whether cell wall anchored SdrC mediates bacterial adherence to Nrx1β-expressing eukaryotic cells. We chose to express both proteins in heterologous systems that do not display any know interaction proteins. The full-length sdrC gene was cloned in pNZ8037 under a nisin inducible promoter. The plasmid was introduced in *L. lactis* for protein expression. Expression of SdrC on the bacterial cell surface was determined using immunofluorescence microscopy (data not shown).The full length *Nrx1*β gene was cloned in pCMV5 to obtain the translational fusion Nrx1β mCherry [26] and a mutant FLAG Nrx1β-mCherry where the SdrC binding site was replaced with a FLAG tag. These vectors were transfected into CHOK1 cells which were allowed to grow for 36 hours to ensure protein expression. Transiently transfected cells were incubated with *L. lactis* transformed with the vector alone, *L. lactis* expressing SdrC, *S. aureus* Newman, *S. aureus* Newman *sdrC::Em^r^* (DU 5988), USA300, MW2, or MRSA252 and bacterial attachment to the cultured mammalian cells were followed. Our results indicated that ten to twenty percent of the microorganisms expressing SdrC adhere to CHOK1 cells expressing wild-type Nrx1β. In contrast, we did not detect attachment when bacteria were incubated with untransfected cells or cells transiently transfected with the inactive FLAG Nrx1β-mCherry or when Nrx1-mCherry expressing cells were incubated with the *S. aureus* mutant DU5988 or *L. lactis* transformed with an empty vector (Fig. 5C). Fluorescence micrographs revealed that bacteria colocalize with Nrx1β (Fig. S4).

## Discussion

The success of *S. aureus* as a pathogen partly depends on the organism ability to effectively colonize different tissues in the host [9],[27], evade host defense systems [28] and resist antibiotic therapy [29]. The molecular mechanisms involved in the development of different staphylococcal infections are incompletely understood but likely involve a large set of virulence factors. Many of these virulence factors interact with and manipulate specific molecular targets in the host. To interact with host targets most virulence factors are either secreted or exposed on the surface of the bacteria. Gram-positive bacteria express a family of surface proteins that are covalently anchored to the cell wall at their C-terminus. The amino acid sequences of these CWA proteins vary dramatically from one organism to another. Nevertheless, structural predictions identify a subgroup of CWA proteins with a common structural organization. This subgroup includes the known staphylococcal MSCRAMMs but also several proteins with unknown function. We hypothesize that the latter also act as adhesins and interact with molecules in the host [7],[9]. In our search for ligands for these orphan adhesins, we here report that screening a phage display library of linear peptides is a useful strategy to indentify host targets for orphan MSCRAMMs. To demonstrate the validity of this approach we first used SdrG_N2N3_ to pan a commercially available library of 12 amino acids inserts in the P3 phage protein. The consensus sequence identified from this analysis overlapped with the previously determined SdrG binding site located in the N-terminus of the Fg β chain. We subsequently predicted a N2N3 domain in SdrC and used a recombinant form of this segment as a target in panning the peptide library. A pattern search using the degenerate consensus sequence returned by the phage display experiment against the whole human proteome identified β-neurexin isoforms as the putative binding partners for SdrC. The human proteome contains three β-neurexin proteins, which share approximately 60% identity with each other [14]. Each β-neurexin isoform contains a variant of the peptide sequence identified by phage display located close to the amino-terminal end of the protein, which is exposed on the surface of the eukaryotic cell and therefore is accessible for binding. Moreover, this sequence starts nine residues after the first amino acid of β-neurexin, which is particularly interesting because the SdrG_N2N3_ binding site in Fg also maps nine residues from the N-terminus of the β chain polypeptide.

Next, we demonstrated that both the SdrC_N2N3_ and the SdrC_A_ fragments bind with high affinity to the amino-terminal segment of Nrx1β, only when the polypeptide contains the amino acid sequence identified by phage display. The interaction does not promote bacterial internalization (Fig. S1), the Kd of interaction is not influenced by the amount of protein coated on the plates (Fig. S2) and it is not influenced by metal ions (Fig. S3). We further demonstrated that a synthetic peptide corresponding to the binding site in β-neurexin effectively inhibits the binding of SdrC to Nrx1β. These data indicated that recombinant SdrC_N2N3_ is able to bind recombinant Nrx1β and that the binding site is the amino acid sequence identified by phage display. In this study, we have also shown that the binding of SdrC to Nrx_wt_ peptide exhibits a K_D_ of 2.5×10^−7^, which is comparable to those recorded for high affinity CWA protein/host protein interactions. Further, we showed that *S. aureus* Newman, USA300, MW2 and MRSA252 release a fragment of SdrC capable of binding Nrx1β. Heterologous expression of SdrC on the surface of *L. lactis* mediates bacterial attachment to Nrx1β transiently expressed on CHOK1 cells. Similarly, clinical strains expressing SdrC adhere to wild-type Nrx1β heterologously expressed in CHOK1. This experiment demonstrates that SdrC can interact with Nrx1β when the two proteins are appropriately expressed on bacteria and mammalian cells, respectively.

The biological significance of the SdrC-Nrx1β interaction is unclear. mRNA for β-neurexins is found in many tissues [30] but the protein was only detected in neuronal tissues. It is possible that Nrx1β protein expression occurs in non-neuronal cells or tissues that have not yet been examined and/or that protein expression in non-neuronal tissues is induced under certain conditions. SdrC may serve as an adhesin mediating bacterial attachment to Nrx1β in staphylococcal infections of neuronal tissues or where the target protein is expressed. It is also possible that during the course of the infection biologically active fragments could be released from the bacteria during proteolytic processing of CWA proteins. SdrC_N2N3_ fragments which are released and resistant to further proteolylic degradation could interact with Nrx1β at tissues distant from the primary infection site. In this scenario it would be interesting to examine if SdrC fragments can induce intracellular signaling through its interaction with Nrx1β or interfere with the neurexin-neuroligin interaction.

A review of the literature reveals that staphylococcal endocarditis and sepsis have been associated with polyneuropathy and in several rare cases even with reversible acute tetraplegia. Studies suggest that polyneuropathy may often follow after sepsis [31],[32],[33]. Before recent advances in medicine, such as life support in the ICUs, septic death likely occurred before the neuromuscular signs could be observed. The most common manifestations of polyneuropathy are difficulty in weaning from the ventilator and limb weakness. Electrophysiologic and histopathologic investigations demonstrated axonal degeneration of motor and sensory fibers that is different from autoimmune syndromes that can develop during infection. The etiology of sepsis-associated polyneuropathy is unclear. It was thought to be caused by neuromuscular blocking agents used to ease mechanical ventilation, antibiotic toxicity, corticosteroids, microanoxia, nutritional deficiency, and high levels of inflammatory mediators. However, the cause of the disease remains unclear and is likely due to a combination of the above-mentioned factors (reviewed in [31]). Moreover, the molecular mechanism of axonal degeneration in sepsis-associated polyneuropathy is unknown. On the other hand, studies investigating neurotransmission, revealed that inhibition of Nrx1β-Nlg interaction led to late-onset neuromuscular deterioration [34]. These observations raise the possibility that SdrC interaction with Nrx1β may contribute to sepsis-associated polyneuropathy. Future studies will investigate in detail the ability of SdrC to interfere with neurexin biology *in vitro* and *in vivo*.

## Materials and Methods

### Media and growth conditions

*E. coli* was cultured in LB medium (Sigma, St. Louis, MO) containing ampicillin (100 µg/ml) at 37°C with shaking at 250 rpm. *S. aureus* was cultured in tryptic soy broth at 37°C with shaking at 250 rpm. *L. lactis* was cultured in GM17 (Oxoid, LTD, Hampshire, UK) containing 0.5% glucose and chloramphenicol (10 µg/ml) (Sigma, St. Louis, MO) at 30°C without shaking. To express SdrC in *L. lactis*, overnight cultures were diluted 1∶100, grown for another 3 hours and induced with nisin (1.6 ng/ml) overnight, unless otherwise mentioned. CHOK1 cells were grown in CD CHO media (GIBCO, Grand Island, NY) at 37°C in a humidified chamber with 5% CO_2_.

### Bacterial strains used for adhesion

*S. aureus* strains MW2, USA300 and MRSA were from NARSA or previously described [35]. *The sdrC* null mutant (*sdrC*::pG^+^Host) of *S.aureus* Newman (DU5988) was described previously [36]. The *sdrC* gene was cloned into the nisin-inducible expression vector pNZ8037 [37] and transferred into *L.lactis* NZ9800 by previously described procedures [38].

### Molecular modeling

Modeling of SdrC was performed using PHYRE **P**rotein **H**omology/analog**Y** **R**ecognition **E**ngine Version 2.0 (http://www.sbg.bio.ic.ac.uk/phyre/html/index.html)

### Plasmid construction

DNA manipulation was performed using standard methods. DNA modification and restriction enzymes were purchased from New England Biolabs, Inc. (Ipswick, MA) or Promega (Madison, WI) and used according to the manufacturer protocol. Fragments encoding different domains of SdrC or Nrx1β were amplified by PCR from *S. aureus* genomic DNA, plasmids pCMV5 Nrx1β-Fc or pCMV5 FLAG-Nrx1β-mCherry [26] and oligonucleotide primers listed in Table 4. The PCR products were analyzed by agarose gel electrophoresis and purified using a QIAquick gel extraction kit (Qiagen, Sciences, MD). To construct SdrC expression plasmids, a *Bam*HI-*Hin*dIII fragment containing the appropriate gene segment was cloned into pQE30 (Qiagen, Sciences, MD). To construct the Nrx1β-mCherry translational fusion, we first amplified the N-terminal 765 nt of Nrx1β from pCMV5 Nrx1β-Fc to obtain a PCR product corresponding to the first 255 aa of neurexin protein (including the signal sequence). Second, we amplified the Nrx1β 255-mCherry from plasmid pCMV5 FLAG-Nrx1β-mCherry to obtain the translational fusion of the 3′ end fragment of neurexin gene with mCherry from *Discosoma* sp. The newly synthesized PCR fragments were used as a template for an overlapping PCR to obtain full length neurexin 1β-mCherry as a translational fusion. A *Bgl*II-*Xba*I fragment containing the overlapping PCR was cloned into pCMV5.

**Table 4:** Oligonucleotide primers used to amplify gene fragments.

Expression construct	Primer sequence
SdrC_52–496_ (SdrC_A_)	5′ CGCAGGATCCGCAGAACATACGAATGGAG
	5′ CGCAAAGCTTACTTTTGGTCGCCATTAGCAG
SdrC_178–496_ (SdrC_N2N3_)	5′CCCGGATCCGGAACAAATGTTAATGATAAAGTACAT
	5′CCCAAGCTTTTATTTCTTTTGGTCGCCATTAG
Nrx_47–255_	5′CACCATGGCATCCAGTTTGGGAGCGC
	5′TTTCACATTTCCCACTATGGCGATG
Nrx_70–255_	5′CACCATGAGTTTGGGAGCGCACCACA
	5′TTTCACATTTCCCACTATGGCGATG
Nrx_1–255_	5′CCGCAGATCTATGTACCAGAGGATGCTCCGGT
	5′TTTCACATTTCCCACTATGGCGATG
Nrx_255-mCherry_	5′CATCGCCATAGTGGGAAATGTGAT
	5′GCGCTCTAGATTACTTGTACAGCTCGTCCATGCCG

The enzyme restriction sites are underlined.

To obtain GST-tagged expression proteins we amplified Nrx_47–255_ (aa) or Nrx_70–255_ (aa) from pCMV5 Nrx1β-Fc using primers described in Table 1. After purification we cloned these fragments in the *E. coli* expression system with Gateway Technology (Invitrogen, Inc, Carlsbad, CA) according to the manufacturer's instructions. All plasmid constructs were sequenced to ensure the integrity of the amplified fragments (Baylor College of Medicine DNA Sequencing Core Facility).

### Protein expression and purification

Plasmids pQE30-SdrC_52–496_ and pQE30-SdrC_178–496_ were transformed into *E. coli* Topp 3. Overnight starter cultures were diluted 1∶50 in LB containing ampicillin (100 µg/ml) and incubated with shaking until the culture reached OD_600_ 0.6–0.8. Protein expression was induced by adding 0.1 mM IPTG (final concentration) and continuing the incubation for 4 hours. Bacterial cells were harvested by centrifugation, resuspended in PBS and frozen at −80°C. Plasmids pDEST Nrx_47–255_ and pDEST Nrx_70–255_ were transformed into BL21-AI (Invitrogen, Inc, Carlsbad, CA). Cultures were induced with 0.1% arabinose for 16 h at room temperature. Bacterial cells were harvested by centrifugation, resuspended in PBS containing EDTA-free Complete Protease Inhibitor (Roche Diagnostics, Mannheim, Germany) and frozen at −80°C.

To purify SdrC_52–496_ and SdrC_178–496_, cells containing recombinant protein fragments were passed through a French press (1100 p.s.i.). Cellular debris was removed by centrifugation at 40,000 rpm for 20 minutes followed by filtration through a 0.45 µM membrane. The filtered bacterial lysate were applied at 2 ml/min on a 5 ml nickel-charged HiTrap Chelating column (GE Healthcare, Uppsala, Sweden) equilibrated with 10 mM Tris HCl, 100 mM NaCl pH 7.9. The column was washed with 40 volumes of 10 mM Tris HCl, 100 mM NaCl, 20 mM imidazole. Bound protein was eluted with a linear gradient of imidazole (10 to 200 mM, total volume 200 ml) yielding proteins that were >95% pure.

Fractions containing the SdrC 52–496 recombinant protein were dialyzed overnight in 4 L of 25 mM Tris-Cl pH 7.9 containing 10 mM EDTA and 1 mM 1,10 O-phenantroline. The dialysed sample was applied at 2 ml/minute to a MonoQ anion exchange column equilibrated with 25 mM Tris-Cl pH 7.9 for further purification. Recombinant protein of interest was collected from the flow through and dialyzed against PBS pH 7.4. Sample was reapplied on a nickel-charged HiTrap Chelating column. Pure protein was dialyzed against 50 mM EDTA to remove excess nickel and then in HBS (10 mM HEPES pH 7.4, 150 mM NaCl, 3 mM EDTA) to remove excess EDTA.

Fractions containing the SdrC_178-499_ recombinant protein were dialyzed overnight in 25 mM Tris-Cl pH 8.7. Dialyzed sample was applied at 2 ml/minute to a MonoQ anion exchange column equilibrated with 25 mM Tris-Cl pH 8.7. Bound protein was eluted with a linear gradient of NaCl (0–1 M NaCl, 160 ml). Fractions containing pure protein were dialyzed against 50 mM EDTA to remove excess nickel and then against HBS to remove excess EDTA.

To purify neurexin GST-tagged fragments, cells were passed through a French press (1100 p.s.i.) in the presence of Complete protease inhibitor tablets (Roche Molecular Biochemicals). Cellular debris was removed as described above. Clear cell lysate was applied to a glutathione-Sepharose column (Sigma, St. Louis, MO). Bound protein was eluted with 10 nM glutathione pH 8. Recombinant protein was concentrated and applied to a S-75 Sephadex (GE Healthcare, Uppsala, Sweden). Proteins were eluted with PBS pH 7.4.

Mass spectrometry analysis and N-terminal sequencing were performed at Tufts Protein Core Facility, Tufts University.

### SDS-PAGE and Western

Recombinant proteins were analyzed by SDS-PAGE using standard procedure [39] on 12% acrylamide gels. Gels were stained with Coomassie Blue. CWA proteins from cells grown to exponential (OD_600_ = 8) or stationary phase (10 h) were harvested, washed 3 times with PBS and concentrated to OD_600_ = 50. CWA proteins were solubilized with 200 µg/ml lysostaphin in a buffer designed to minimize bacterial lysis composed of 30% sucrose, 50 mM Tris/Base pH 8, 20 mM MgCl_2_ and 50 µg/ml protease inhibitor (Roche) for 30 min at 37°C. Cell wall fractions were then incubated with 100 µl Sepharore-IgG for 3 h at 4°C to remove protein A. Culture supernatants were concentrated to OD_600_ = 10 after the addition of 100 µg/ml protease inhibitor (Roche). Protein A was removed by incubating with 100 µl Sepharore-IgG. Extracts or supernatants were separated on 4–15% SDS-PAGE gels. For Western blotting, proteins were transferred electrophoretically to nitrocellulose or polyvinylidene difluoride membranes (Bio-Rad, Hercules, CA) using the semi-dry system (Bio-Rad, Hercules, CA) in Tris (0.02 M)-Glycine (0.15 M)-methanol (20%) buffer. Membranes were blocked in 10% milk and incubated with the appropriate serum. Briefly, membranes A1, A2, B1 and B2 were incubated with rabbit anti-SdrC_N2N3_ serum (H.T.I. BioProducts, Inc) and the appropriate secondary serum, which is listed below. B2 membrane was stripped (Restore western blot stripping Buffer, Pierce, Rockford, IL) for 10 minutes at room temperature and then re-probed with a rabbit anti-SdrC B-repeats serum (H.T.I. BioProducts, Inc) (B3). The membrane was stripped a second time and incubated for 1 hour at room temperature with Nrx_47–255_ recombinant protein, followed by goat anti-GST serum. (Invitrogen, Carlsbad, CA) (B4). A control membrane identical with B2 was probed with the same sera but was not incubated with Nrx_47–255_ recombinant protein (B5). HRP-labeled goat antibody (1∶5000) or HRP-labeled goat anti-mouse or HRP-labeled rabbit anti-goat (BioRad, Hercules, CA) were used to detect bound primary antibody by incubating for 1 hour at room temperature. Membranes were developed with ECL reagent (Pierce, Rockford, IL), exposed to Kodak X-ray film.

### Phage display

NEB Ph.D.12-mer random library was incubated with immobilized SdrC N2N3, SdrG N2N3 or BSA at 1 µg/well. The input was 1.5×10^11^ transducing units with a complexity of 2.7×10^9^ electroporated sequences. The assay was performed according to the manufacturer's instructions. The output phage plaques were 10^4^ transducing units. Wells were washed with 0.5% Tween in TBS buffer, blocked with 10% BSA and incubated with 1×10^8^ transducing units of each amplified phage or insertless phage (fd-tet). To remove unbound phage, wells were washed 10 times with 0.5% Tween in TBS. Bound phage was detected with anti-M13-HRP antibody (GE Healthcare, Buckinghamshire, UK).

### Solid phase binding assay

Each well of Immulon 4BH plates was coated with 1 µg of recombinant Nrx_47–255_-GST, Nrx_70–255_-GSTor GST overnight at 4°C. Coated wells were blocked for 1 hour at room temperature with 2% BSA in 0.05% Tween-TBS buffer. Increasing concentrations of SdrC_N2N3_ or A-region were added to the wells and incubated for 1 hour at room temperature. Bound protein was detected with a polyclonal antibody (1∶3000) against SdrC_N2N3_ (H.T.I. BioProducts) followed by an anti-rabbit HRP-labeled antibody (1∶5000) (Bio-Rad Labs, Hercules, CA). Color development was performed using Sigma*Fast* OPD (Sigma, St. Louis, MO) and the binding was measured using a microtiter plate reader (Molecular Devices) at 450 nm. For inhibition assays, 0.5 µM SdrC_178-496_ protein was incubated with increasing concentrations of peptides prior to the solid phase binding assay. Data presented represent the mean±SD of three independent experiments performed in triplicate. The binding was analyzed by non-linear regression for one binding site (GraphPad Prism).

### Fluorescence polarization

Nrx_wt_ and Nrx_scr_ peptides were labeled with fluorescein as described previously [10]. Increasing concentrations of SdrC_178-496_ were incubated with 10 nM labeled peptide for 3 hours in the dark at room temperature. Polarization was measured using Luminescence Spectrometer LB50B (Perkin Elmer). The data were analyzed by non-linear regression for one binding site. The equilibrium dissociation constant was calculated using the equation ΔP = ΔP_max_ x [protein])/(K_D_ + [protein]) (Eq.1) where ΔP is the change in fluorescence polarization, ΔP_max_ is the maximum change in fluorescence polarization and K_D_ is the equilibrium dissociation constant of the interaction.

For inhibition assays, 0.5 µM SdrC_178–496_ was first incubated with increasing concentrations of unlabeled Nrx_wt_ or unlabeled Nrx_scr_ for 3 hours at room temperature. The mixture was then incubated for 3 hours with fluorescein-labeled Nrx_wt_. The results presented represent the mean±SD of three independent experiments.

### Adherence assay

CHOK1 cells were grown in 24-well tissue culture plates to approximately 80% confluence. Cells were transfected with either pCMV5 Nrx1β-mCherry or pCMV5 FLAG-Nrx1β-mCherry using Lipofectamine 2000 (Invitrogen, Carlsbad, CA) according to the manufacturer's instructions and grown for 36 hours to allow for protein expression. For attachment assays, bacteria were washed 2 times in PBS, resuspended in CD CHO media and incubated for 1 h with CHOK1 cells at an MOI of 10. To remove unattached bacteria, cells were washed 4 times with PBS. To determine the number of attached bacteria, CHOK1 cells were lysed with sterile water and serial dilutions plated on the appropriate media. The number of attached bacteria was reported as a percent of total bacteria at the end of the incubation period. Each experiment was performed in triplicate wells and repeated three times. Statistical analysis was performed using the Student's t-test.

### Protein accession numbers (UniProt) SdrC

*S. aureus* Newman - O86487; *S. aureus* USA300 - A8YZQ9; *S. aureus* MW2 - Q8NXX7; *S. aureus* MRSA252 - Q6GJA7. Neurexin1β: P58400.

## Supporting Information

Table S1 Pattern search results
(0.06 MB PDF)

Figure S1 Thirty-six hours prior to infection, CHOK1 cells where transfected with Nrx1β-mCherry. Bacteria were added to each well at MOI of 10 and incubated for 3 hours in a humidified incubation chamber at 37°C with 5% CO_2_. To remove unbound bacteria, wells were washed 3 times with PBS. After washing, cells were incubated in DMEM containing gentamicin (100 µg/ml) for 1 h. Cells were washed 3 times in PBS, lysed with water and dilution plated. Internalized bacteria where represented as percent of total bacteria at the end of the incubation period.
(0.10 MB PDF)

Figure S2 The solid phase binding assay was performed as described in Materials and Methods. The wells were coated with 0.5 µg/well Nrx_47–255_.
(0.10 MB PDF)

Figure S3 The solid phase binding assay was performed as described in Materials and Methods. The wells were coated with 1 µg/well Nrx_47–255_. EDTA, [Ca2+], [Mg2+], [Mn2+] were added to 1 µM SdrC_N2N3_ prior incubation with Nrx1β at a final concentration of 10 µM.
(0.10 MB PDF)

Figure S4 CHOK1 cells transiently transfected with Nrx1β-mCherry or FLAG Nrx1β-mCherry and grown on glass coverslips were incubated with either *L. lactis* empty vector, *L. lactis* SdrC, *S. aureus* Newman or DU5988 (*S. aureus* Newman *sdrC*::Emr) Nrx1β expression was monitored by the red fluorescence due to mCherry. Bacterial cells were detected with anti-SdrC antibodies and FITC-labeled secondary antibodies (green). CHOK1 nuclei were stained with DAPI (blue). Attachment of bacteria to Nrx1β-expressing CHOK1 cells is shown (merged).
(2.28 MB PDF)
