# Supplementary material for: β-Neurexin Is a Ligand for the Staphylococcus aureus MSCRAMM SdrC
Source: PLoS Pathog. 2010 Jan 15;6(1):e1000726. doi: 10.1371/journal.ppat.1000726 (PMC2800189; doi:10.1371/journal.ppat.1000726)
Supplement: Table S1 — Pattern search results (0.06 MB PDF) [file ppat.1000726.s001.pdf]

Supplementary tables

Table S1. Pattern search results

a. Zero mismatch

Protein	Accession number	Sequence	Predicted localization of the sequence
Neurexin 1 beta	NP_620072	AHHIHHFHG	extracellular

b. One mismatch

Protein	Accession number	Sequence	Predicted localization of the sequence
-	NP_620072	AHHIHHFHG	extracellular
Neurexin 2 beta	NP_620063	THHVHHFHS	extracellular

c. Two mismatches

Protein	Accession number	Sequence	Predicted localization of the sequence
Neurexin 1 beta	NP_620072	AHHIHHFHG	extracellular
Neurexin 2 beta	NP_620063	THHVHHFHS	extracellular
Neurexin 3 beta	NP_620063	QHEHHFHG	extracellular
Voltage dependent T-type calcium channel alpha 1I subunit	NP_66919.2	AHHIHHG	extracellular
