# Supplementary figures and images for: β-Neurexin Is a Ligand for the Staphylococcus aureus MSCRAMM SdrC
Source: PLoS Pathog. 2010 Jan 15;6(1):e1000726. doi: 10.1371/journal.ppat.1000726 (PMC2800189; doi:10.1371/journal.ppat.1000726)

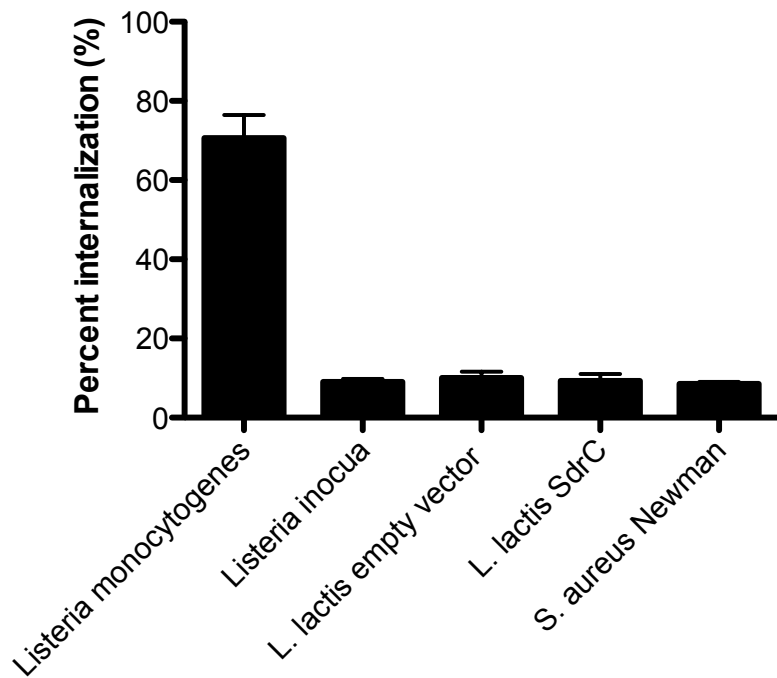

Supplement: Figure S1 — Thirty-six hours prior to infection, CHOK1 cells where transfected with Nrx1β-mCherry. Bacteria were added to each well at MOI of 10 and incubated for 3 hours in a humidified incubation chamber at 37°C with 5% CO2. To remove unbound bacteria, wells were washed 3 times with PBS. After washing, cells were incubated in DMEM containing gentamicin (100 µg/ml) for 1 h. Cells were washed 3 times in PBS, lysed with water and dilution plated. Internalized bacteria where represented as percent of total bacteria at the end of the incubation period. (0.10 MB PDF) [file ppat.1000726.s002.pdf]

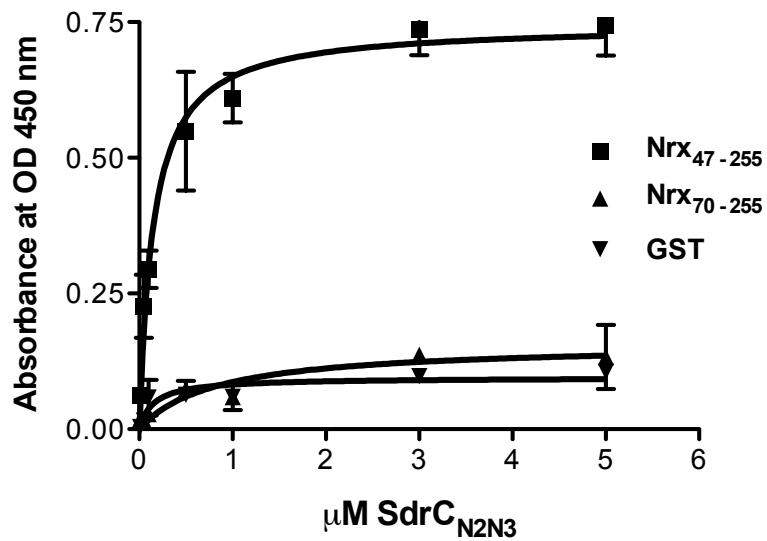

Supplement: Figure S2 — The solid phase binding assay was performed as described in Materials and Methods. The wells were coated with 0.5 µg/well Nrx47–255. (0.10 MB PDF) [file ppat.1000726.s003.pdf]

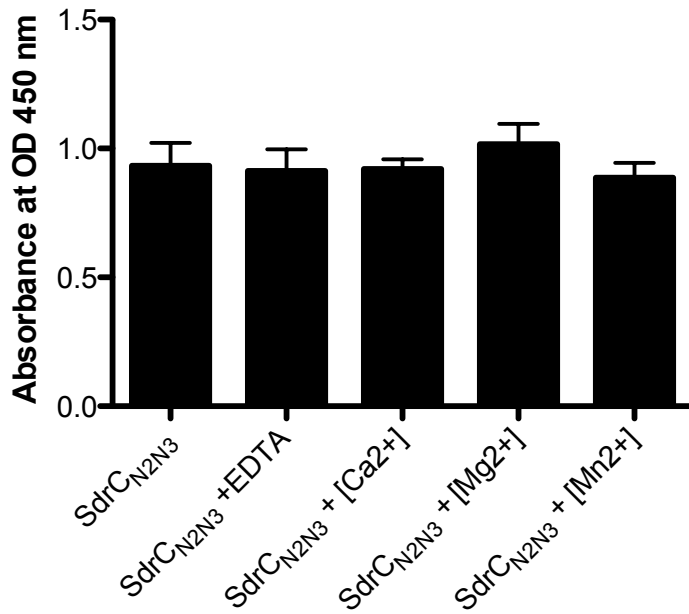

Supplement: Figure S3 — The solid phase binding assay was performed as described in Materials and Methods. The wells were coated with 1 µg/well Nrx47–255. EDTA, [Ca2+], [Mg2+], [Mn2+] were added to 1 µM SdrCN2N3 prior incubation with Nrx1β at a final concentration of 10 µM. (0.10 MB PDF) [file ppat.1000726.s004.pdf]

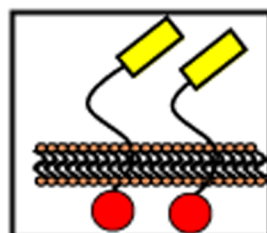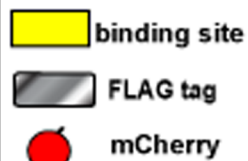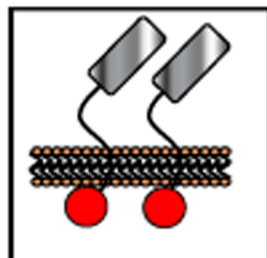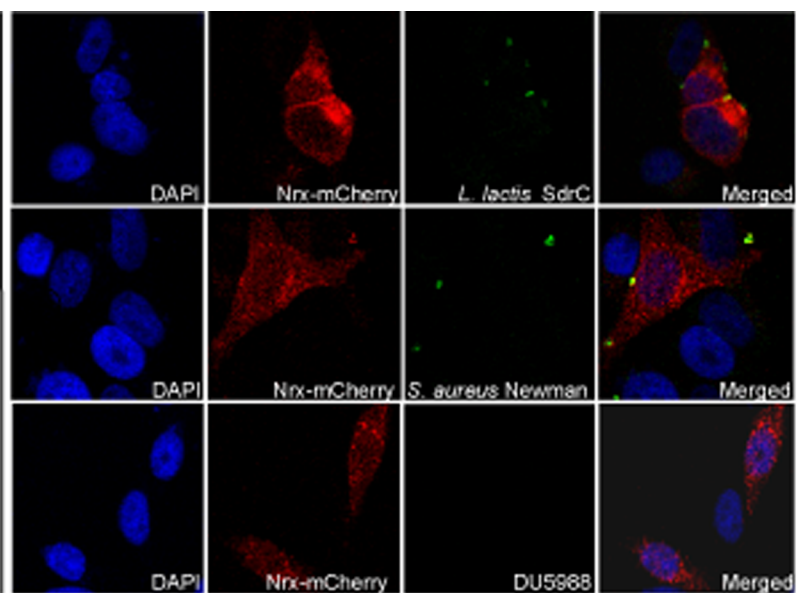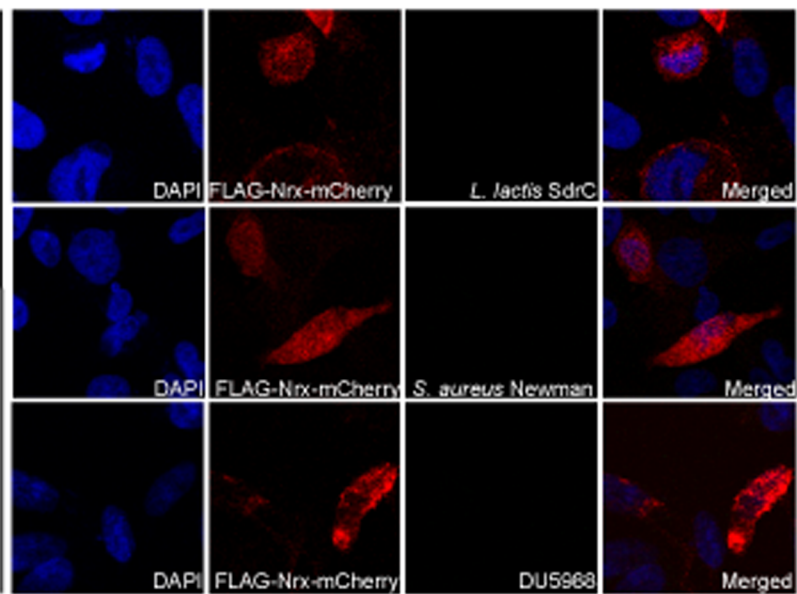

Supplement: Figure S4 — CHOK1 cells transiently transfected with Nrx1β-mCherry or FLAG Nrx1β-mCherry and grown on glass coverslips were incubated with either L. lactis empty vector, L. lactis SdrC, S. aureus Newman or DU5988 (S. aureus Newman sdrC::Emr) Nrx1β expression was monitored by the red fluorescence due to mCherry. Bacterial cells were detected with anti-SdrC antibodies and FITC-labeled secondary antibodies (green). CHOK1 nuclei were stained with DAPI (blue). Attachment of bacteria to Nrx1β-expressing CHOK1 cells is shown (merged). (2.28 MB PDF) [file ppat.1000726.s005.pdf]
